# Reliability and validity of the death anxiety scale in patients with mental illnesses in a general hospital

**DOI:** 10.3389/fpsyt.2026.1881533

**Published:** 2026-09-29

**Authors:** Yanping Duan, Boheng Zhu, Wenqi Geng, Jinya Cao, Bindong Dai, Dandan Ma, Weixuan Qu, Jing Jiang, Tao Li, Jing Wei

**Affiliations:** Department of Psychological Medicine, Peking Union Medical College Hospital, Chinese Academy of Medical Sciences & Peking Union Medical College, Beijing, China

**Keywords:** death anxiety, general hospital, mental illnesses, reliability, validity

## Abstract

**Background:**

Death anxiety is a transdiagnostic construct of mental illness. The aim of this study was to explore the reliability and validity of the death anxiety scale (DAS) in the context of mental illnesses in a general hospital.

**Methods:**

Patients with generalized anxiety disorder, panic disorder, major depressive disorder, obsessive–compulsive disorder or somatic symptom disorder were included in the study. Internal consistency was explored using Cronbach’s α. Construct validity was assessed by exploratory factor analysis (EFA), exploratory graph analysis (EGA) and confirmatory factor analysis (CFA). Scales such as the 17-item Hamilton Depression Rating Scale (HAMD-17), the Hamilton Anxiety Scale (HAMA), the 7-item Generalized Anxiety Disorder Scale (GAD-7), the Somatic Symptom Disorder-B Criteria Scale 12 (SSD-12), the State-Trait Anxiety Inventory (STAI), and the 20-item Toronto Alexithymia Scale (TAS-20) were used to assess the discriminant validity of the DAS.

**Results:**

A total of 585 patients are enrolled in the study. The Cronbach’s α coefficient for the total scale is 0.944. The EFA results in two-factor structures. However, CFA fit indices show poor model fit. The 2-factor model identified by EGA may offer a better fit (χ²/df=5.244, CFI = 0.956, TLI = 0.939, IFI = 0.956, NFI = 0.946, RMSEA = 0.102), comprising cognitive death anxiety and emotional death anxiety. Compared with the HAMD-17, HAMA, GAD-7, SSD-12, STAI, and TAS-20, the DAS has good discriminant validity.

**Conclusion:**

The DAS has good reliability when applied to common mental illnesses in a general hospital. EGA seems to be the best fitting method for exploring the characteristics of death anxiety among patients with mental illnesses in a general hospital. Death anxiety can be categorized into emotional death anxiety and cognitive death anxiety. Although the model achieves an acceptable goodness of fit, it still has limitations, and the characteristics of death anxiety among patients need to be further explored.

## 1 Introduction

Fear of death is a common psychological experience among humans, encompassing a complex array of negative emotional responses—including unease, worry, and distress—that emerge when individuals confront the inevitability and uncontrollability of death (1). In general hospitals, the fear of death represents a prevalent psychological phenomenon among patients dealing with physical illnesses and/or comorbid mental disorders (2). From both existential philosophical and psychoanalytic theoretical perspectives, fear of death constitutes a meaningful topic worthy of in-depth investigation. A better understanding and appropriate management of death anxiety may help clinicians provide more tailored and effective care for patients (3).

Death anxiety has been conceptualized as a transdiagnostic construct underlying a wide range of mental disorders (4, 5). Previous research has identified death anxiety as a robust predictor of psychopathology, including the number of lifetime diagnoses, psychotropic medication use, hospitalizations, subjective distress and functional impairment, depression, anxiety, and perceived stress (6). Some findings have also demonstrated that fear of death exacerbates compulsive washing (7), social avoidance (8), and excessive bodily checking and hypervigilance towards physical symptoms (9). Notably, Iverach et al. argued that current gold-standard treatments for mental health conditions often fail to target the underlying fear of death (10). However, the aforementioned studies are all based on the understanding at the symptomatic level, and are not grounded on the basis of clear psychiatric diagnosis. Common mental disorders among outpatient populations in general hospitals include generalized anxiety disorder (GAD), panic disorder (PD), major depressive disorder (MDD), obsessive–compulsive disorder (OCD), and somatic symptom disorder (SSD). Current understanding of these diseases remains insufficient.

The conceptualization and measurement of death anxiety remain important issues that require further exploration. Early studies investigated the conceptual structure of death anxiety using open-ended self-report questionnaires (11). From the perspective of cognitive, existential, and psychoanalytic frameworks, death anxiety has been linked to fear of dying, loss of meaning, and fear of separation. In 1970, Templer developed the 15-item self-reported death anxiety scale (12). With increasing research attention, subsequent studies by Templer (13) and other investigators (14–16) have demonstrated that the DAS exhibits a diverse factor structure, that may vary with sample source and size. In China, researchers developed and validated a revised DAS to assess four components of death anxiety: dysphoria, death intrusion, fear of death, and death avoidance. These four dimensions reflect somatic, cognitive, emotional, and behavioural manifestations of death anxiety (17), and the scale has demonstrated good reliability and validity among young people.

However, it remains unclear how to use the DAS in patients with mental illnesses in China. Accordingly, the present study adopted the DAS developed within the Chinese cultural context (17). We administered the DAS to patients with various mental disorders, including GAD, PD, MDD, OCD, and SSD, to assess the reliability and validity of the DAS in a general hospital.

## 2 Materials and methods

### 2.1 Participants

This study was conducted as part of the National Multi-center Research Project of Science and Technology Innovation in China 2030: “Research on Pathogenesis and Intervention Techniques of Anxiety Disorder” (Project No.: 2021ZD0202001). On the basis of the data and framework of this national project, our study further evaluated the reliability and validity of the DAS among patients with common mental disorders in a general hospital.

The inclusion criteria were as follows: (1) Aged 18 to 60 years; (2) Meeting the Diagnostic and Statistical Manual of Mental Disorders, Fifth Edition (DSM-5) diagnostic criteria for GAD, PD, MDD, OCD, or SSD; and (3) provided written informed consent.

The exclusion criteria were as follows: (1) a history of or current severe mental illnesses, including neurodevelopmental disorders, schizophrenia spectrum disorders, bipolar disorder spectrum disorders, psychoactive substance use-related disorders, or neurocognitive disorders; (2) a history of major neurological or physical diseases, including cardiovascular, cerebrovascular respiratory, digestive, urinary, immune, haematological, infectious diseases, or malignant tumours; (3) being pregnant or lactating; (4) receiving modified electroconvulsive therapy (MECT) or electroconvulsive therapy (ECT) within the past 6 months, or currently receiving hormone therapy; (5) having immediate family members with a history of bipolar disorder; (6) having hearing impairment or colour blindness; and (7) having other circumstances deemed inappropriate for study participation.

### 2.2 Measures

To evaluate the validity of the DAS, several criterion scales were employed, including the 17-item Hamilton Depression Rating Scale (HAMD-17), the Hamilton Anxiety Scale (HAMA), the 7-item Generalized Anxiety Disorder Scale (GAD-7), the 12-item Somatic Symptom Disorder-B Criteria Scale (SSD-12), the State-Trait Anxiety Inventory (STAI), and the 20-item Toronto Alexithymia Scale (TAS-20).

#### 2.2.1 DAS

The DAS, originally developed by researchers from the Research Centre of Brain Function and Psychological Science, Shenzhen University, has previously undergone reliability and validity testing in Chinese youth populations, with a four-factor structure comprising dysphoria, death intrusion, fear of death, and death avoidance. Specifically, this scale is designed to assess death anxiety across somatic, cognitive, emotional, and behavioural dimensions; from the symptomatic perspective of one’s own death, it measures dysphoria and somatic components, general emotional and behavioural components specific to death anxiety, and intrusive thoughts related to death (17).

#### 2.2.2 Validation instruments

The HAMD-17 was administered by professionally trained psychiatrists to assess depressive symptoms. This scale has demonstrated good reliability and validity and has been widely used in clinical research (18). It can reflect the severity of depressive symptoms, with cut-off scores of 7, 17, and 24, which represent mild, moderate, and severe depressive symptoms, respectively.

The HAMA is widely used in clinical practice to assess the severity of anxiety symptoms. A cut-off score of 14 points was adopted in the present study, with a total score exceeding this threshold indicating the presence of anxiety symptoms (19).

The GAD-7 is a self-report questionnaire designed to assess anxiety symptoms. This scale has been validated for reliability and validity as a tool for measuring anxiety in the general population (20). The GAD-7 scoring criteria are as follows: scores of 0–4 points indicate no or extremely mild anxiety; scores of 5–9 points indicate mild anxiety; scores of 10–14 points indicate moderate anxiety; and scores of 15–21 points indicate severe anxiety.

The SSD-12 was developed based on the DSM-5 diagnostic criteria for Somatic Symptom Disorder (SSD)—Criteria B. Each psychological sub-criterion was assessed using four items, with all items scored on a 0–4 scale. In Chinese populations, previous studies have identified a cut-off score of 16 or 17 for SSD-12 to detect SSD (21).

The STAI comprises state anxiety and trait anxiety (22). State anxiety is defined as an unpleasant emotional experience accompanied by autonomic nervous system overactivity, which is typically transient. In contrast, trait anxiety represents a relatively stable anxiety tendency characterized as a personality trait with individual differences. The STAI has a cumulative score range of 20 to 80, with a minimum score of 20 and a maximum score of 80. This scale is sensitive to anxiety symptoms and is reliable for analysing symptoms in patients with panic attacks, generalized anxiety disorder, and obsessive–compulsive disorder (23).

The TAS-20 is a reliable and valid instrument that accurately reflects and measures the construct of alexithymia, which is characterized by deficits in affect awareness, expression, and operational thinking (24). The TAS-20 consists of three subscales, namely, difficulty identifying feelings, difficulty describing feelings, and externally oriented thinking (25).

### 2.3 Statistical analysis

Descriptive statistics: Continuous variables were reported as mean ± standard deviation (mean ± SD) if the data conformed to a normal distribution, and as median (minimum, maximum) otherwise. For categorical variables, counts and percentages are presented.

Reliability analysis: Cronbach’s reliability coefficient α was used to assess the internal consistency reliability of the total score, each domain, and subtotal. Additionally, the Cronbach α coefficient was calculated for each item removed to ensure that this did not result in an increase in any instance. Split-half reliability was determined by calculating Guttman’s split-half coefficient. Cronbach’s α coefficients greater than 0.70 were considered sufficient (26).

Item analysis: Corrected item–total correlations were calculated to measure the strength of the relationship between each item and the total score of the scale.

Construct validity: Exploratory factor analysis (EFA) and criterion-related correlation analysis of the data were conducted using SPSS 26.0 statistical software. Confirmatory factor analysis (CFA) was performed using AMOS 23.0. The sample was randomly split in 1/3 to perform exploratory graph analysis (EGA) (27) using R version 4.3.2 and the package EGAnet version 1.1.1 (28). After EFA and EGA explored the factor model, CFA was conducted to confirm the underlying construct. Several indices were adopted to evaluate model fit, including χ²/df, Comparative Fit Index (CFI), Tucker–Lewis Index (TLI), Incremental Fit Index (IFI), Normed Fit Index (NFI) and Root Mean Square Error of Approximation (RMSEA). The cutoff criteria were as follows: χ²/df < 5 (acceptable) and < 3 (good); CFI, TLI, IFI and NFI> 0.90 (acceptable) and > 0.95 (excellent); RMSEA < 0.08 (acceptable) and < 0.06 (good).

Criterion-related validity was evaluated by employing the HAMD-17, HAMA, GAD-7, SSD-12, STAI, and TAS-20 as criterion measures. Pearson’s correlation coefficients (r) were calculated to assess the associations between the factor scores of the death anxiety scale (DAS) and the total scores of each criterion scale.

The significance level was set as bilateral P<0.05.

## 3 Results

### 3.1 Characteristics of the sample

A total of 585 patients were included in the study, including 204 patients with GAD (34.9%), 126 with PD (21.5%), 133 with MDD (22.7%), 42 with OCD (7.2%), and 80 with SSD (13.7%). There were 381 females (65.1%) and 204 males (34.9%). The average age was 35.70 ± 9.31 years (**Table 1**).

**Table 1:** Characteristics of the participants (n=585).

Item	Subitem	N	%
Gender	Female	381	65.1%
	Male	204	34.9%
Age (years)	Mean ± SD	35.70 ± 9.31	
Ethnicity	Han	555	94.9%
	Others	22	3.8%
	missing data	8	1.3%
Marital Status	Unmarried	219	37.4%
	Married	329	56.2%
	Divorced	21	3.6%
	Widowed	1	0.2%
	Others	15	2.6%
Education	Primary school	6	1.0%
	Junior middle school	32	5.5%
	High school	37	6.3%
	Technical Secondary School	89	15.2%
	Undergraduate degree	276	47.2%
	Master’s degree	124	21.2%
	Doctor degree	9	1.5%
	Missing data	12	2.1%
Residence	Countryside	89	15.2%
	Cities	482	82.4%
	Others	14	2.4%
Diagnosis	GAD	204	34.9%
	PD	126	21.5%
	MDD	133	22.7%
	OCD	42	7.2%
	SSD	80	13.7%

GAD, generalized anxiety disorder; PD, panic disorder; MDD, major depressive disorder; OCD, obsessive–compulsive disorder; SSD, somatic symptom disorder.

### 3.2 Reliability analysis

Internal consistency reliability: The Cronbach’s α coefficient for the total scale was 0.944. Additionally, after sequentially deleting each item from the DAS, the Cronbach’s α coefficient for the total scale ranged from 0.939 to 0.946.

Item analysis: The corrected item–total correlation ranged from 0.439 to 0.820 (**Table 2**).

**Table 2:** The correlation coefficient between each item and the total score of the DAS.

Item	Item-total correlation (Pearson r)	Item	Item-total correlation (Pearson r)
1	0.712	10	0.622
2	0.439	11	0.720
3	0.725	12	0.722
4	0.611	13	0.780
5	0.709	14	0.820
6	0.633	15	0.748
7	0.573	16	0.770
8	0.534	17	0.750
9	0.796		

Split-half reliability: The correlation coefficient of odd and even items indicated that the unequal-length Spearman–Brown coefficient was 0.905.

### 3.3 Construct validity

The total sample was split into two independent sample sets. The first subsample (n=179) was used to conduct Exploratory Factor Analysis (EFA) and Exploratory Graph Analysis (EGA) to explore the potential factor structure of the scale. The second independent subsample (n=406) was applied to perform Confirmatory Factor Analysis (CFA) to cross-validate the factor structure derived from the exploratory analyses.

EFA: We employed the EFA to analyze the data of the discovery set samples.The KMO value was 0.924, and Bartlett’s test of sphericity was statistically significant (χ² = 2570.680, p < 0.001). Principal axis analysis and direct oblimin analysis was performed. The factor loading for item 2 is 0.164, which is less than 0.40. After eliminating items with factor loading less than 0.40, or items with factor loading on both factors greater than 0.350, all remaining items had factor loading above 0.50. The cumulative variance contribution rate was 67.730%. A two-factor model was ultimately developed comprising the following items: Factor 1 consists of Items 4, 6, 11, 13, 14, 16 and 17, indicating Cognitive and behavioral death anxiety; Factor 2 consists of Items 7, 8, 10 and 15, indicating emotional death anxiety (**Table 3**).

With a fixed number of factors set at 4, the KMO value was 0.924, and Bartlett’s test of sphericity was statistically significant (χ² = 2570.680, p < 0.001). The cumulative variance contribution rate was 73.998%. When constructing the measurement model, the factor loadings of items 2 and 9 are less than 0.40. The KMO value was 0.914, and Bartlett’s test of sphericity was statistically significant (χ² = 2368.753, p < 0.001). The cumulative variance contribution rate was 74.245%. After deleting these two items, the resulting 4-factor model was: Factor 1 consists of Items 11, 12, 13, 14, 16, and 17, indicating dysphoria; Factor 2 consists of Items 7 and 8, indicating fear of death; Factor 3 consists of Items 1, 3, 5, and 10, indicating death intrusion; and Factor 4 consists of Items 4 and 6, indicating avoidance of death (**Table 3**).

**Table 3:** EFA factor analysis.

Item	2-factor model		4-factor model revised			
	1	2	1	2	3	4
Item 1	–	–	0.021	0.013	**-0.813**	0.119
Item 2	–	–	–	–	**-**	–
Item 3	–	–	0.022	0.058	**-0.721**	0.209
Item 4	**0.767**	-0.105	0.052	0.030	-0.047	**0.798**
Item 5	–	–	0.038	0.037	**-0.616**	0.218
Item 6	**0.832**	-0.094	0.123	0.025	-0.057	**0.798**
Item 7	-0.057	**0.948**	0.038	**0.880**	-0.078	-0.010
Item 8	-0.099	**0.834**	-0.046	**0.870**	0.008	0.042
Item 9	–	–	–	–	–	–
Item 10	0.158	**0.530**	0.051	0.103	**-0.699**	-0.149
Item 11	**0.883**	-0.042	**0.861**	-0.136	-0.041	0.059
Item 12	–	–	**0.550**	0.088	-0.318	-0.071
Item 13	**0.791**	0.169	**0.898**	0.187	0.180	0.061
Item 14	**0.821**	0.130	**0.845**	0.023	-0.044	0.031
Item 15	0.344	**0.580**	0.315	0.262	-**0.403**	-0.057
Item 16	**0.888**	0.011	**0.857**	-0.039	0.016	0.101
Item 17	**0.730**	0.182	**0.630**	-0.027	-0.261	0.053

Bold formatting indicates that the factor loading in EFA is higher than the factor loadings on other factors

EGA: We also employed the EGA to analyze the data of the discovery set samples. The 2-factor model was the most suitable. After deleting items 3,6,7, factor 1 contained 1, 5, 8, 10, 15; Factor 2 contained 2, 4, 9, 11, 12, 13, 14, 16, 17 (See **Figure 1**).

**Figure 1:**
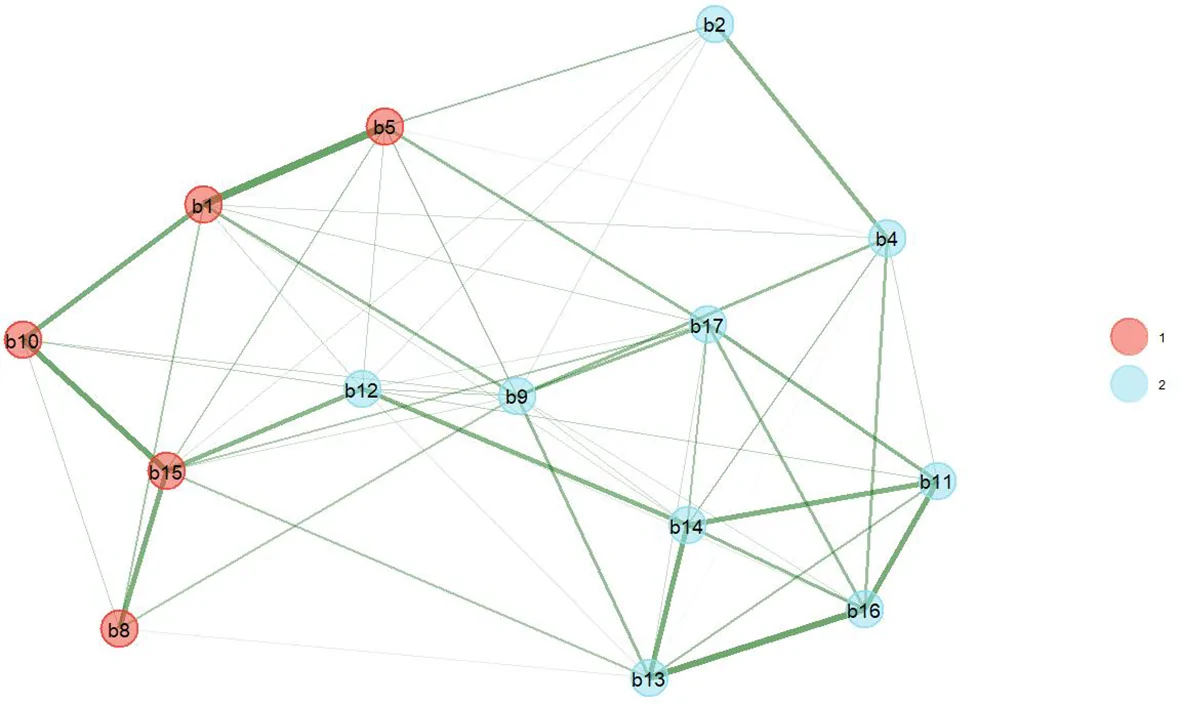
Exploratory Graph Analysis (EGA) approach to detecting communities of the DAS. As observed in this EGA, a total of two distinct latent communities were identified.

According to the EGA repeated verification analysis results, among factors 2, the factor load of items 2, 4, 9, 12 and 17 were lower than 0.7. After deleting these items, the 2-factor model was finally obtained, which was: factor 1 (items 1, 5, 8, 10, 15) indicating cognitive death anxiety, factor 2 (item 11, 13, 14, 16) indicating emotional death anxiety. See **Figure 2** for details.

**Figure 2:**
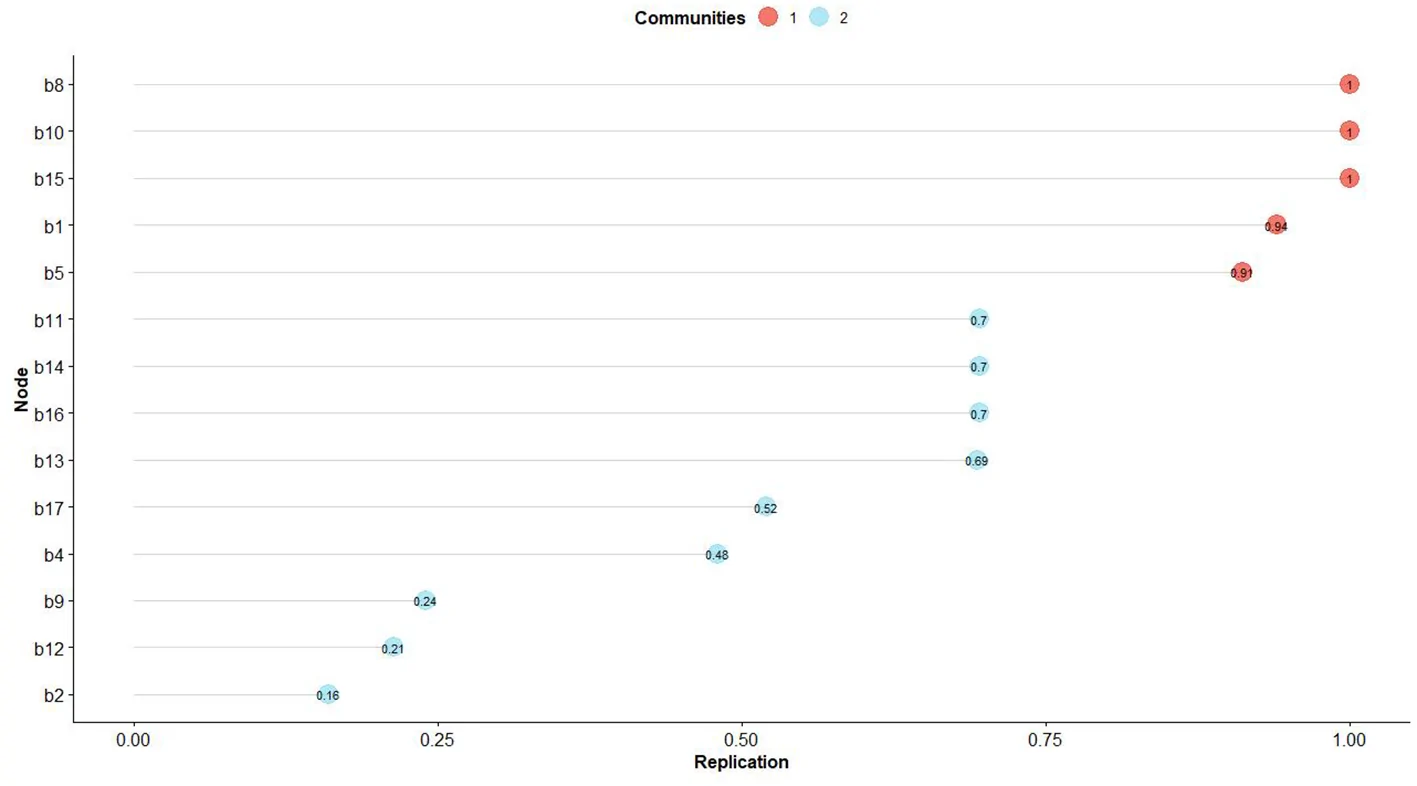
Exploratory Graph Analysis (EGA) repeated validation factor analysis results showed that the 2-factor model was the most suitable, in which factor 1 was composed of item 1, 5, 8, 10, 15, indicating cognitive death anxiety. Factor 2 consists of entries 11, 13, 14, 16, representing emotional death anxiety.

CFA: CFA was performed on an independent sample of 406 participants to assess the fit of four candidate models. These comprised two 2-factor models derived from EFA and EGA, respectively; one 4-factor model based on EFA; and a 4-factor model adapted from Cai et al. (17). The results showed that compared to the other models, 2-factor model had a better fit (χ²/df=5.244, CFI = 0.956, TLI = 0.939, IFI = 0.956, NFI = 0.946, RMSEA = 0.102) (**Table 4**).

**Table 4:** Goodness-of-fit indices of the CFA model for the DAS.

	χ²/df	CFI	TLI	IFI	NFI	RMSEA
2-factor model^a^	10.643	0.866	0.828	0.866	0.855	0.154
2-factor model^b^	5.244	0.956	0.939	0.956	0.946	0.102
4-factor model^c^	6.194	0.903	0.879	0.904	0.887	0.113
4-factor model^d^	6.654	0.873	0.847	0.873	0.854	0.118

a the 2-foctor model constructed via EFA; ^b^the 2-factor model constructed via EGA; ^c^the 4-factor model constructed via EFA; ^d^the 4-factor model constructed the published work of Cai et al. (17).

### 3.4 Criterion-related validity

Using the HAMD-17, HAMA, GAD-7, SSD-12, STAI, and TAS-20 as benchmarks, the Pearson r coefficient was calculated between the factors of the DAS and the benchmarks. The results revealed that the total DAS score was moderately correlated with the GAD-7, SSD-12, and STAI-T scores (**Table 5**).

**Table 5:** Criterion-related validity of the DAS.

Scale	DAS total score	2-factor model	
		Cognitive death anxiety	Emotional death anxiety
HAMD	0.302^**^	0.402^**^	0.144^**^
HAMA	0.342^**^	0.351^**^	0.268^**^
GAD-7	0.397^**^	0.395^**^	0.323^**^
SSD12	0.427^**^	0.326^**^	0.448^**^
STAI-S	0.394^**^	0.426^**^	0.286^**^
STAI-T	0.406^**^	0.456^**^	0.278^**^
TAS	0.223^**^	0.289^**^	0.116^**^

DAS, death anxiety scale; HAMA, Hamilton Anxiety Scale; HAMD17, Hamilton Depression Rating Scale-17 items; GAD-7,7-item Generalized Anxiety Disorder Scale; SSD12, Somatic Symptom Disorder-B Criteria Scale 12; STAI-S, State-Trait Anxiety Inventory-State; STAI-T, State-Trait Anxiety Inventory-Trait; TAS, Toronto Alexithymia Scale.

**P<0.001.

## 4 Discussion

In the reliability analysis results of this study, the Cronbach’s α coefficients of the internal consistency reliability analysis was all above 0.9, indicating that the reliability of this scale is extremely good. The correlation coefficients between each item of the DAS and the total score were above 0.4, indicating that each item has good discrimination and can be retained. The split-half reliability is good, and the internal consistency is reliable. These results are consistent with previous study (17).

During the construction of the factor model via EFA based on principal axes, items with factor loadings below 0.40, or items with factor loadings greater than 0.350 on two factors, which indicate cross-loading, need to be deleted. EFA can explore whether the factor structure of the DAS is consistent between the patient population and the general population by specifying a fixed number of factors. However, the fit of both EFA-derived factor models was poor in CFA. The model derived from EGA has a better model fit than that derived from EFA, which may be attributed to the following reasons: First, the overlapping variance between dimensions of the DAS interferes with the estimation of factor loadings, and the pattern matrix from oblique rotation cannot completely eliminate this interference, whereas EGA uses partial correlations to achieve clearer item clustering. Second, EFA allows for greater degrees of freedom in subjective researcher decision-making, as the factor retention criteria, the loading threshold of 0.4, and the exclusion rule for cross-loadings of 0.35 are all artificially set. By contrast, EGA performs one-step dimension identification, which reduces structural deviations caused by multiple rounds of artificial screening. Our results support the previous researches (32) in defining EGA as a more accurate method. For the EGA fitting indices, such as CFI = 0.956, TLI = 0.939, IFI = 0.956, and NFI = 0.946, which indicate that the model fit is acceptable. However, RMSEA = 0.102, which is higher than the threshold of 0.08, and χ²/df = 5.244, which exceeds the cut-off value of 5, suggesting that the model still has shortcomings. This issue may be related to the wording of items in the scale. For example, the item that can be categorized into the cognitive death anxiety dimension uses the word “feel”, which is likely to cause confusion among respondents and thus leads to collinearity effects. During scale revision, changing “feel” to “think” may help better distinguish between different factors.

In previous studies, death fear has been defined as fear associated with life meaning, separation, death and other related contents (11). Some scholars also posit that death fear is linked to anxiety related to freedom, loneliness, life meaning and other constructs (31). The present study conducts a thematic analysis of death anxiety among patients diagnosed with GAD, PD, MDD, OCD, SSD and other related disorders. Based on symptom items, Cognitive death anxiety refers to concerns related to death-related thoughts, imagery, dreams, the meaning of life and the connection with reality. This dimension includes item 1 “I often think about the scene of my own death”, item 5 “I often feel that I will die soon”, item 8 “I often dream about death-related things”, item 10 “When I think about death, I often feel that my life is meaningless”, and item 15 “When I think about death, I often feel a sense of strangeness or alienation from others”. Emotional death anxiety refers to death-related fear, somatic responses, dysphoria, panic. This dimension includes the following items: item 11 “When I think about death, I often feel afraid”, item 13 “When I think about death, my heart often beats rapidly”, item 14 “When I think about death, I often feel mentally disturbed”, and item 16 “When I think about death, I often feel panicked”. Understanding death anxiety among patients with mental disorders from the perspectives of cognition and emotion may be simpler and clearer than analyzing it from the four aspects including somatic, cognitive, emotional, and behavioral aspects (17). In this study, the symptom items 5, 10, and 15 can be conceptualized as cognitive death anxiety. However, the use of “feel” in the item description may confuse respondents, potentially introduce multicollinearity, and thereby reduce the fit indices of CFA. Adopting the wording “think” for item description may improve the validity of this dimension.

In this study, when HAMD-17, HAMA, GAD-7, SSD-12, STAI, and TAS-20 were used as reference validity indicators for calculating correlation validity, a moderate correlation was detected between dysphoria and SSD-12, and a moderate correlation was detected between fear of death and the STAI. Compared with the above scales, the discriminant validity of the DAS was good. This conclusion can enhance the understanding of death-related fear among clinicians and scientists. Previous studies have suggested that death anxiety might be related to the defence mechanisms of depression, genetic predisposition, early childhood adverse events, personality traits, insecure attachment, absence of meaning, trauma, stress, and life events (10) Fear of death may also be related to religion (29). Gender may also affect fear of death; for example, previous studies have suggested that compared with men, women might be more prone to experiencing death anxiety (30). Some studies have also suggested that death anxiety is a transdiagnostic trait (10). This study also supports that fear of death may be a transdiagnostic characteristic.

In conclusion, this study revealed that the DAS has good reliability and validity and that the 2-factor model applied by EGA is more applicable to a general hospital. This study has the following limitations: 1, The research subjects were patients with GAD, PD, OCD, SSD and MDD who visited the outpatient department of a general hospital. Fear of death is also commonly observed among individuals with conditions including post-traumatic stress disorder (33), somatic disorders characterized by prominent health anxiety (2), alcohol use disorder (34) and eating disorders (35). These types of mental disorders were not included in our study. Specifically, the number of patients attending general hospital outpatient clinics with post-traumatic stress disorder, eating disorders and alcohol use disorder is relatively small, while somatic disorders characterized by prominent health anxiety have already been analyzed in previous studies. For these reasons, these patient groups were not included in this study. The application scope of DAS in general hospitals still needs further improvement. 2, Unequal sample sizes were observed across groups. A portion of the participants were sourced from the baseline data of the study *Research on Individualized and Pathway-Based Psychosomatic Integrative Therapy for Anxiety Disorders Based on Brain Science* (ChinCTR2300074416). Consequently, Patients with GAD and PD are more willing to participate in questionnaire surveys, resulting in a relatively larger proportion of GAD and PD patients in the sample. MDD is more prevalent among outpatient populations. The sample sizes for patients with OCD and SSD are relatively small, which is associated with the lower willingness of this patient population to participate in research. Accordingly, the characteristics of the DAS explored in this study may have limitations when applied to interpret patients with OCD or SSD. Separate analyses for OCD and SSD patients are required in future research. 3, The reference standards selected were related to various dimensions of anxiety, subjective and objective depression symptoms and emotional expression, but related scales such as personality, life events and growth experiences were not included. Further exploration of the fear of death can aid in understanding more assessment scales of psychological dimensions as reference tools to evaluate the use of the DAS.

## 5 Conclusion

The DAS has good reliability when applied to common mental illnesses in a general hospital. EGA seems to be the best fitting method for exploring the characteristics of death anxiety among patients with mental illnesses in a general hospital (χ²/df=5.244, CFI = 0.956, TLI = 0.939, IFI = 0.956, NFI = 0.946, RMSEA = 0.102). Death anxiety can be categorized into emotional death anxiety and cognitive death anxiety. Although the 2-factor model of EGA achieves an acceptable goodness of fit, it still has limitations, and the characteristics of death anxiety among patients need to be further explored in future studies.

## Data Availability

The data analyzed in this study is subject to the following licenses/restrictions: <b>The datasets used in the study can be available from the corresponding author on reasonable request. Requests to access these datasets should be directed to duanyanping@pumch.cn.
